# Developing public health ethics learning modules – can we learn from critical pedagogy?

**DOI:** 10.1186/s40985-015-0006-z

**Published:** 2015-08-28

**Authors:** Jutta Lindert, Christopher Potter

**Affiliations:** 1 University of Emden, Emden, Germany; 2 Brandeis University, Waltham, USA; 3 Cardiff University, Cardiff, UK

**Keywords:** Ethics, Modules, Critical pedagogy

## Abstract

Curriculum development in masters of public health programs that effectively meets the complex ethical challenges of the 21st century is an important part of public health education and requires ethical reasoning and purposively thinking. Current master programs in Public Health do not regularly include modules in public health ethics. The aim of this paper is to present background and theoretical foundation for developing a methodology and methods to develop public health ethics modules for curriculum development in public health programs. We describe ethical reasoning in medicine and in Public Health, specify pedagogical approaches and key assignments organized around the critical pedagogy.

## Introduction

When asked to write a review on methodology to develop Public Health ethics modules we immediately thought among others about ethical challenges in the 21st century such as the commercial creation and sale of embryos, child abuse, domestic violence, elder abuse, wars and terrorism and health disparities. Are Public Health students prepared for these Public Health threats be traditional Public Health contents and traditional conventional pedagogy?

Much work has been done in the past two decades to develop theories of Public Health ethics [1–5]. However, a series of articles indicated that the teaching of ethics in Schools of Public Health is still relatively limited [6]. Only in some schools of Public Health in the United States (e.g. in the Harvard School of Public Health, the School of Public Health, University of North Carolina at Chapel Hill), and the United Kingdom (e.g. at the London School for Hygiene and Tropical Medicine), ethics modules in Public Health are offered. This chapter recognizes that many countries have national qualifications frameworks for Public Health and that each institution has its own realities and needs. Yet, we will discuss a theoretical framework for developing Public Health ethics modules (contents and pedagogy).

## Review

### Paulo Freire and critical pedagogy theory

There are a variety of models for the design of modules in Bachelor, Master and PhD courses and many of the same issues are relevant in the context of designing ethics modules such as definition of aims, agreement on learning outcomes and resources, learning and teaching strategies, assessment criteria and evaluation. In the context of ethics modules in Public Health the tradition of critical pedagogy can take a prominent place.

The central idea of the critical pedagogy is a philosophy of education and social movement that combines education with critical theory. First described by Paulo Freire [7, 8], it has since been further developed by others such as Stage, Muller, Kinzie, and Simmons as a praxis oriented educational movement: “Education’s role is to challenge inequality and dominant myths…Learning is directed towards social change” [9]. As Freire argued, “Students, as they are increasingly posed with problems relating to themselves in the world and with the world, will feel increasingly challenged, and obliged to respond to that challenge…the resulting comprehension tends to be increasingly critical…Their response to the challenge evokes new challenges, followed by new understandings; and gradually the students come to regard themselves as committed” [10]. Freire viewed both research and education as venues where power operates.

This means that in the critical pedagogy approach the locus of the learning process is shifted from the teacher to the students utilizing learner-centered methods of enriching the educational content of teaching processes through joint decision making and collective learning. Thus, applying critical pedagogy in the development of Public health ethics modules can build on students’ knowledge, foster critical dialogue and analysis.

## Ethical analysis in Public Health

In this section we outline major approaches to ethical analysis. Three approaches have shaped ethical reasoning in general, medical ethics and Public Health ethics. Ethical traditions have been shaped mainly by the deontological, consequentialist and virtue based approaches [11–13]. Such traditions or paradigms have been extensively considered for the field of medical or clinical practice, but only comparatively recently have the significant differences and challenges within public health (with its collectivist focus on safeguarding the community and promoting well-being) been appreciated [14].

For Public Health ethics it is important first to come to some agreement of what is meant by *public health*. How it is defined and what is its content–mission, functions, and services? Definitions for health and for Public Health vary widely [15]. Health can be defined *inter alia* as absence of diseases, as well-being [16], or as the means to (or resources for, or foundations of) achieving goals [17]. Herewith, the point of Public Health is to protect and improve the *health of populations* and to engage social justice. Related to this focus on groups are the following definitions of public Health: “*Public Health is what we, as a society, do collectively to assure the conditions of people to be healthy*” [18] and “*Public Health involves not only traditional government actions to protect the public from imminent danger, but also, at a more fundamental level, cooperative behavior and relationships of trust in communities, as well as a far reaching agenda to address complex social, behavioral and environmental conditions that affect health*” [18].

In contrast to some other sciences more focused on creating new knowledge, Public Health practitioners know what they are hoping to achieve: the improvement in *population* health not in health of individuals [5, 19]. By public health ethics we mean understanding how to evaluate different courses of action and non-action and their individual and social consequences. As such, public health ethics embraces the range of methods used to analyze, interpret and evaluate the variety of ways individuals and groups interact with each other in a framework of ethics. Herewith, ethics refers to the way of evaluating, reflecting, understanding and criticizing what constitutes moral life and actions and manifests itself in reflections and case studies in Public Health [11]. Herewith development of modules in teaching public health ethics build on students’ knowledge to experience ethical modules as something they do, not as something done to them.

Generally, ethical reasoning seeks to provide an account of how humans assign and evaluate the rightness or wrongness of arguments and their consequences. Additionally, ethics is related to interpersonal duties and obligations that are not regulated or compelled by external authority. As indicated above, in the past (and sometimes still today) ethical reasoning in public health has been approached as a sub-set of medical ethics [14, 20–22].

Following this application of ethical reasoning, it is necessary to distinguish between applied ethics and ethical theories in public health. Descriptive ethics relates to accounts of how humans behave, normative ethics considers what should be done. Both approaches have a long tradition in medical ethics.

Medical ethics in health care has a long tradition, and has focused on the dyadic clinical relationships between medical doctors and patients and a set of issues related to such relationships (e.g. autonomy, beneficence, non-maleficence, justice, consent, confidentiality [13]. The most common environment for such reasoning is the hospital where patients seek medical care. In those situations, the patient is in a vulnerable state, seeking relief from a problem. The clinician is in a role of authority which is based largely on their knowledge and their resources. Such, there is thus an imbalance of power between the patient and the clinician. This imbalance includes power imbalance in terms both of knowledge and power to act. The key principles of medical ethics are therefore related to such situation: regulating the potential abuses of power between clinicians and patients; between one who holds most of the authority and resources, and the other who is vulnerable in part because of his or her illness. Because of the orientation towards relationships in medical ethics, the concepts of autonomy and the’ negative rights’ of the person (e.g. the right not to be harmed) have tended to dominate in this field of medical ethics [23].

Taken together, traditional medical ethics has an occupation with distributive justice at the micro-level, to the neglect of broader social and global justice issues. In contrast, public health presents and creates distinctive ethical challenges, not just problems already familiar from medical ethics because it is embedded by definition in a population perspective [21, 24–26]. As a consequence of the population perspective, public health sometimes promises benefits for population groups at the cost of benefits for individuals [24]. This may create tension between the rights and individuals and the rights and needs of populations [27].

Additionally, in contrast to clinical medicine which uses medical interventions to cure or treat existing illness, public health uses primarily non-medical means such as infrastructure improvement, policy, law and behavioral health to prevent illness. This aspect means that Public health ethics needs a broader framework to analyze competing interests.

## Application of the critical pedagogy to development of ethics modules in Public Health

Several Public Health ethics frameworks have been developed, which were divided into practice–based, mostly deriving from several or no philosophical schools, and theory-based frameworks, mostly based on one philosophical school [15, 28]. Most notably, Beauchamp [11] Bayer’s New Ethics for the Public’s Health Beauchamp [2] and Childress’s Public Health Law and Ethics [29] were the first edited collections on the subject. Since then, however, there have been numerous books and scholarly articles on which scholars can now draw and Oxford Journals launched a journal called *Public Health Ethics* in 2008. We will briefly summarize approaches to teaching Public Health ethics.

First, *deontological* approaches in Public Health ethics, following Kant’s ‘categorical imperative’ argue that human beings ought to be treated with respect, as ends in themselves, not as means to another individual’s ends [25]. According to these deontological approach, ethical acts are appraised independently of the consequences that follow from them [28]. This ethical theory is closely aligned with accounts of human dignity and inherent worth. This is different in consequentialist theories [29]. In contrast, within *consequentialist* theories the rightness or wrongness of actions is looked at in terms of the consequences that results from these actions. For consequentialist, the rightness of an action is not intrinsic, but is determined by adding up the pleasure and pain it produced. The classical, widely used formulation of consequentialism is known as *utilitarianism*, where the best action is that which created the greatest good for the greatest number [30, 31]. Utilitarians are divided into subjective utilitarians and objective utilitarians. Subjective utilitarians believe that wellbeing is best defined by each individual’s personal experience, objective utilitarians want to centralize the assessment process. Utilitarianist approaches are prominent in Public Health ethics. These are often associated with economic analyses such as cost-benefit and cost-effectiveness analyses.

Rather than focusing on acts themselves or on consequences, *virtue*-*based* approaches in ethics examine the characteristics of actions of humans [30]. As such they identify qualities of behavior and focus on characteristics such as courage, humility, caring and wisdom [32]. Further approach is the Social justice bases approach. In this approach social justice itself is a virtue. However, as many of the values of Public Health are virtue based this approach has its own attraction for Public Health teaching at the moment. We can identify at least two applications of the virtue based approach: one view is that every community defines its own norms (relativist communitarianism), by contrast, universalist communitarians, believe in a single true form of good society and its associated virtues. [33, 34] Knowledge about these main approaches to ethics may serve to enhance self-reflection by students, and researchers and help them to be aware of existing guidelines and codes of conduct for public health [35].

The need for developing specific modules in Public Health ethics led to a meeting to form a Public Health Ethics Interest Group (PHEIG) in 2001. PHEIG had two main goals: the first was to develop a curriculum in Public Health ethics at the PhD level and the second was to host an international symposium on Public Health Ethics in 2002. In this symposium four key thematic areas were identified: surveillance and regulation, individual rights versus collective rights, risk and precaution and global health equity. However, in this symposium it became clear that there are further approaches within public health ethics (Table 1) which are useful for further development of Public health ethics [35].

**Table 1:** Goals of public health ethics modules

1	Stimulating the moral imagination (including the ability to gain a feel for the lives of others)
2	Recognizing ethical problems
3	Gathering of facts
4	Developing analytical skills
3	Ethical priorisation
4	Identification of conflicting ethical values
5	Application of ethical standards
6	Looking for further ways to resolve the conflict

Additionally to the reasoning on approaches codes of ethic were developed. Codes of ethics are aspirational statements of expected qualities and behaviors of public health experts. In 2000, the graduating class of the Public Health Leadership Institute in the United States drafted a code for ethics as a group project, which is available at http://www.ncbi.nlm.nih.gov/pmc/articles/PMC1447186/ [36]. This code is based on 12 ethical principles and 11 values and beliefs that underlie these principles. The consensus reached is seen in Table 2 [37].

**Table 2:** Principles of the ethical practice of public health

1	Public Health should address principally the fundamental cause of disease and requirements for health, aiming to prevent adverse health outcomes.
2	Public Health should achieve community health in a way that respects the rights of individuals in the community.
3	Public health policies, programs, and priorities should be developed and evaluated through processes that ensure an opportunity for input from community members.
4	Public Health should advocate for, or work for the empowerment of, disenfranchised community members, ensuring that the basic conditions necessary for health are accessible to all people in the community.
5	Public Health should seek information needed to implement effective policies and programs that protect and promote health.
6	Public health institutions should provide communities with the information they have that is needed for decisions on policies or programs and should obtain the community’s consent for implementation.
7	Public health institutions should act in a timely manner on the information they have within the resources and mandate given to them by the biblical.
8	Public health programs and policies should incorporate a variety of approaches that anticipate and respect diverse values, beliefs, and cultures in the community.
9	Public health programs and policies should be implemented in a manner that most enhances the physical and social environment.
10	Public health institutions should protect the confidentiality of information that can bring harm to an individual or community if made public. Exceptions must be justified on the basis of the high likelihood or significant harm to the individual or others.
11	Public health institutions should ensure the professional competence of their employees.
12	Public health institutions and their employees should engage in collaborations and affiliations in ways that build the public’s trust and the institution’s effectiveness.

Source: American Public Health Association. American Public Health Association: public health code of ethics available at: http://nnphi.org [37]

## Principles in teaching public health ethics

One of the first issues students may need to confront is the problem of objectivity in Public health and the socially constructed nature of our understanding of public health threats. Similarly, whether a public health situation requires ethical attention, why and how, will be the result of competing perspectives. Ethics analysis accepts that Public Health science is value related and often power dominated. Real world examples in the form of case studies can contribute to generate critical thinking of students in Public Health ethics (See the article by Christopher Potter in this series). These case studies [38] may enable students to become critical subjects in the classroom and assume responsibility for the public health in the sense of the critical pedagogy: to communicate effectively, to act as advocates, to encourage and lead change, and to understand and balance competing needs and rights. This leads to a re-thinking of the challenge of power in public health. Many public health dilemmas are backed by power: power of those who define the ethical problem.

A commonly used definition of power comes from Max Weber, who defined power as the ability to influence others or events by force, rewards, favors and resources. Public health by definition is focused on populations, and on regulation and public policy. This adds to the peculiarity of public health ethics: the exercise of public authority and the imposition of public sanctions and penalties in an area as deeply personal as an individual’s health [1, 5]. As such access to power and how power is used means treating people as means lies at the heart of the development of Public Health ethics modules.

What is recognized as important for Public Health ethics and who defines the importance? Who defines what is important? Which possibilities are available to safeguard that individual people are not used as a means rather than an end–either as research participants or as research colleagues, or those who will be exposed to harm while safeguarding the wider population? These questions need further investigation and reasoning. One starting point for any public health practitioner analyzing the *status quo* or addressing a situation that appears to require change is to ask *Qui bono*? Who benefits? This simple question can begin to lay open the system that has brought about the situation, suggest ways of resolving it (including data collection and communication strategies), and provide a framework for assessing competing interpretations and suggested solutions.

## Conclusions

Developing modules in ethics for Public Health with a critical pedagogy approach calls for faculty able to train in “real-world” applications of ethical dilemmas. Establishing ethics in Public health in situations of power, is a challenge. The theories outlined in this paper provide opportunities of ethical reasoning in public health by investigating threats to Public health in a human rights framework as regards content and methods.
